# Establishing complexity targets to enhance artificial reef designs

**DOI:** 10.1038/s41598-024-72227-z

**Published:** 2024-09-27

**Authors:** Elisabeth Riera, Benjamin Mauroy, Patrice Francour, Cédric Hubas

**Affiliations:** 1 Université Côte d’Azur, CNRS, ECOSEAS, Parc Valrose, 06108 Nice Cedex 02, France; 2 Muséum National d’Histoire Naturelle, UMR 8067 BOREA, MNHN-SU-CNRS-UCN-UA-IRD, Station Marine de Concarneau, Concarneau, France; 3 Université Côte d’Azur, CNRS, UMR 7351 LJAD, Parc Valrose, 06108 Nice Cedex 02, France

**Keywords:** Artificial reef, Habitat complexity, 3-Dimensional computer-aided design model, Marine biology, Ecosystem ecology, Restoration ecology, Urban ecology, Ecology

## Abstract

Artificial reefs (AR), which are integral tools for fish management, ecological reconciliation and restoration efforts, require non-polluting materials and intricate designs that mimic natural habitats. Despite their three-dimensional complexity, current designs nowadays rely on empirical methods that lack standardised pre-immersion assessment. To improve ecosystem integration, we propose to evaluate 3-dimensional Computer-aided Design (3D CAD) models using a method inspired by functional ecology principles. Based on existing metrics, we assess geometric (C-convexity, P-packing, D-fractal dimension) and informational complexity (R-specific richness, H- diversity, J-evenness). Applying these metrics to different reefs constructed for habitat protection, biomass production and bio-mimicry purposes, we identify potential complexity target points (CTPs). This method provides a framework for improving the effectiveness of artificial reef design by allowing for the adjustment of structural properties. These CTPs represent the first step in enhancing AR designs. We can refine them by evaluating complexity metrics derived from 3D reconstructions of natural habitats to advance bio-mimicry efforts. In situ, post-immersion studies can help make the CTPs more specific for certain species of interest by exploring complexity-diversity or complexity-species distribution relationships at the artificial reef scale.

## Introduction

Among the artificial structures spread across the ocean, artificial reefs (AR) can be defined as “submerged structures placed on the seabed deliberately to mimic some characteristics of natural habitats”^1^. The use of artificial reefs made of rocks, wood or bamboo by fishermen dates back at least 3000 years in the Mediterranean^2^. A similar practice has been documented in Japan since the seventeenth century^3^. Over time, these handcrafted practices have been developed on a larger scale using objects from their immediate environment. Recycled materials, such as shipwrecks, offshore platforms, construction waste, and used tyres, were favoured, with no regard for the environmental impacts^4,5^. During the 1970s and 1980s, specific programs for fisheries management were developed on the impulse of the first International Conference on Artificial Reefs and Related Aquatic Habitats (CARAH)^1,6^.

Finally, in the late 2000s, the United Nations Environment Programme published the first guidelines, establishing a precise framework for artificial reef deployment and enlarging their objectives to fish production, habitat protection, habitat restoration and/or regeneration, and recreational opportunities. Nowadays, artificial reefs have to be made from non-polluting inert materials and designed with a structural complexity that mimics the natural habitats of the location^7,8^.

Despite establishing these guidelines, there is still a lack of scientific basis to monitor and compare the effectiveness of such structures^9^. To evaluate the quality and theoretical adequacy of the structure before immersion, precise information is needed regarding the material and design of the reefs. Some studies have investigated the effect of different materials on the primary and macrofouling communities that settle on the artificial reef to select the most suitable substrates according to objectives^10–12^. As far as three-dimensional structure is concerned, artificial reefs are mainly designed empirically based on expert recommendations by quantifying the number of spaces, voids and crevices to assess fish preference for different types of shelter^13^. Since the early 90s, most of the structures used have been simple in shape and have been aggregated randomly underwater without offering much heterogeneity. Assuming that habitat complexity strongly influences the diversity and abundance of species colonizing artificial reefs^5,14–20^; some studies have practiced post-complexification of artificial reefs to improve their effectiveness^5,15,21^. More recently, large scale 3D printing has given rise to a new generation of artificial reefs that more closely mimic the structural complexity of natural habitats^22,23^. A few studies have attempted to use surface roughness^24,25^ or fractal dimension^26,27^ as indicators of the structural complexity of artificial reefs. However, no standardized method is available for assessing the structure of artificial reefs prior to immersion or evaluating their effectiveness based on complexity aspects.

The link between the complexity of the habitat and species diversity is a pillar of functional ecology. In natural ecosystems, a myriad of studies has been published since the studies of MacArthur and MacArthur^28,29^, who proposed that the structural complexity or heterogeneity of the habitat influences the diversity of bird species in an area. The idea that habitat structure can affect species diversity is based on the notion that different species have different ecological requirements and may prefer or require different habitats for survival, reproduction, and resource use. Habitats with greater structural complexity can provide a broader range of ecological niches or opportunities for species with different ecological needs, leading to higher species richness and diversity^30–34^, functional diversity^35^, and higher prey-predator dynamics^36,37^.

Although there is a consensus on the existence of this link, the definition and methods for evaluating it remain debated. Mc Coy and Bell^31^ defined the habitat structure by three different aspects, namely scale, complexity, and heterogeneity, which are closely related to the shape of the structures and the abundance, diversity and arrangement of the structural elements that compose the habitat. The metrics used to assess complexity and heterogeneity can vary according to the scale of the study; this scale dependency can bring high variability between studies and must be precisely defined. This definition has been followed for decades in the literature^34,36,38^. Therefore, the metrics that evaluate habitat structure are classified into two categories. To name the most famous: fractal dimension, rugosity, or vertical relief fall into the complexity category that evaluates the global shape; whereas diversity, richness, or standard deviation fall into the heterogeneity that evaluates the variation of elements in the shape. More recently, Loke and Chisholm^39^ proposed to define complexity and heterogeneity by geometric and informational complexity, respectively and gave recommendations for choosing the most suitable metrics and ensuring comparability between studies. This framework provides a valuable tool to help advance research in these areas, and we will use their categorization hereafter to describe complexity. They also expressed stringent criticisms and limitations on the use of some geometric complexity metrics in favor of informational complexity metrics. However, as Madin and colleagues^40^, we agree that well-defined geometric complexity metrics are relevant for highlighting important ecological responses. Moreover, we believe that no metrics prevail over others if they assess different parameters of the habitat structure related to ecological responses.

Facing these heated debates, we have been cautious in evaluating both geometric and informational complexity of the structure of artificial reefs designed by 3D computer-aided design (CAD). We chose six different measures: fractal dimension (D), Packing (P) and Convexity (C) (as proxies of geometric complexity); and richness (R), diversity (H) and evenness (J) (as proxies of informational complexity). To summarize the overall complexity of the artificial reefs, we combined these six measures into an additional metric called the Complexity Index (CI) to illustrate the global complexity. We then used the six metrics to evaluate various artificial reefs built for different purposes (habitat protection, biomass production and biodiversity enhancement) produced by moulding or 3D printing. First, this approach allowed us to confirm the a priori categorization of the artificial reefs and revealed that each purpose is associated with a specific set of complexity factors. Secondly, we have identified Complexity Target Points (CTPs) that effectively summarize the most complex structure evaluated.

Our method can enhance the effectiveness of artificial reef design by providing a clear understanding of AR structural properties that can be adjusted according to CTPs prior to immersion. Additionally, it provides a quantitative approach to examining the relationship between habitat complexity and diversity of biotic assemblages at the scale of artificial reefs, allowing verification of CTPs’ accuracy post-immersion.

## Materials and methods

### Complexity assessment of 3D CAD models

#### 3D CAD models of artificial reef modules

Our methodology was developed using 3D computer-aided design (CAD) models to generate functional virtual prototypes of three-dimensional artificial reefs. We used STL files, which describe a 3D model's surface using a series of connected triangles defined by their normal vectors and vertices in a 3D Cartesian coordinate system. Our analysis included a range of artificial reef unit models of a volume ranging from 2.35e+05 to 1.19e+07 cm^3^, produced by moulding and 3D printing. The models originated from various sources: some were provided by constructors, while others were modelled on Tinkercad^®^ using dimensions and shapes collected from Tessier and colleagues’ review^5^. Each design was built for specific objectives. Therefore, our selection consists of six designs for habitat protection from illegal trawling, seven designs for biomass production for artisanal fishing support, and seven bio-mimicry designs to enhance biodiversity. Detailed information about the artificial reefs is available in the supplementary materials (Supplementary Table S1).

#### Geometric complexity

An organism needs a specific volume when mobile or a surface when sessile. Therefore, to assess these parameters quantitatively, we were inspired by the metrics "Packing" (P) and "Convexity" (C) from Zunic and Rosin^41^. These metrics help evaluate parameters associated with the volume and surface of the 3D CAD model and its convex hull (the smallest possible convex shape that completely contains the 3D model, with no concave areas). However, we adapted the formulas to our aims. Specifically, P is based on the surface ratio of the convex hull to the 3D CAD model. For C, instead of using the volume of the structure that is inaccessible to mobile organisms, we used the accessible volume available within the convex hull.

Furthermore, to encompass the multiscale structure of the artificial reef models, we used the fractal dimension (D), a widely recognised metric in natural environment complexity analysis that defines how an object fills space at all scales^34,36,39,40^.

#### Informational complexity

To welcome a rich trophic network, an artificial reef must display heterogenous microhabitats^42–44^. In 3D CAD models, we have access to the normal vectors, which vary across the model surface and represent the heterogeneity of the structure. In our study, we refer to this as informational complexity. Ecologically, high surface heterogeneity orientations imply various anchor points promoting sessile species settlement^45–48^. Moreover, it increases the likelihood of creating cavities or shelters attracting a diverse range of mobile species to the artificial reef^5,13,17^. Therefore, each normal vector in the 3D CAD model serves as a parameter representing the heterogeneity of the orientation of the surface in 3D space. We used metrics commonly applied in ecology to determine specific richness (R)^49^, diversity (H)^50^, and evenness (J)^51^ of the normal vectors of the evaluated structures. These metrics provide insights into normal vector orientations in terms of relative distribution, diversity, and homogeneity, which we referred to as 'Orientation Richness,' 'Orientation Diversity,' and 'Orientation Evenness,' respectively.

#### Extraction of parameters and computation of the metrics

**Fig. 1:**
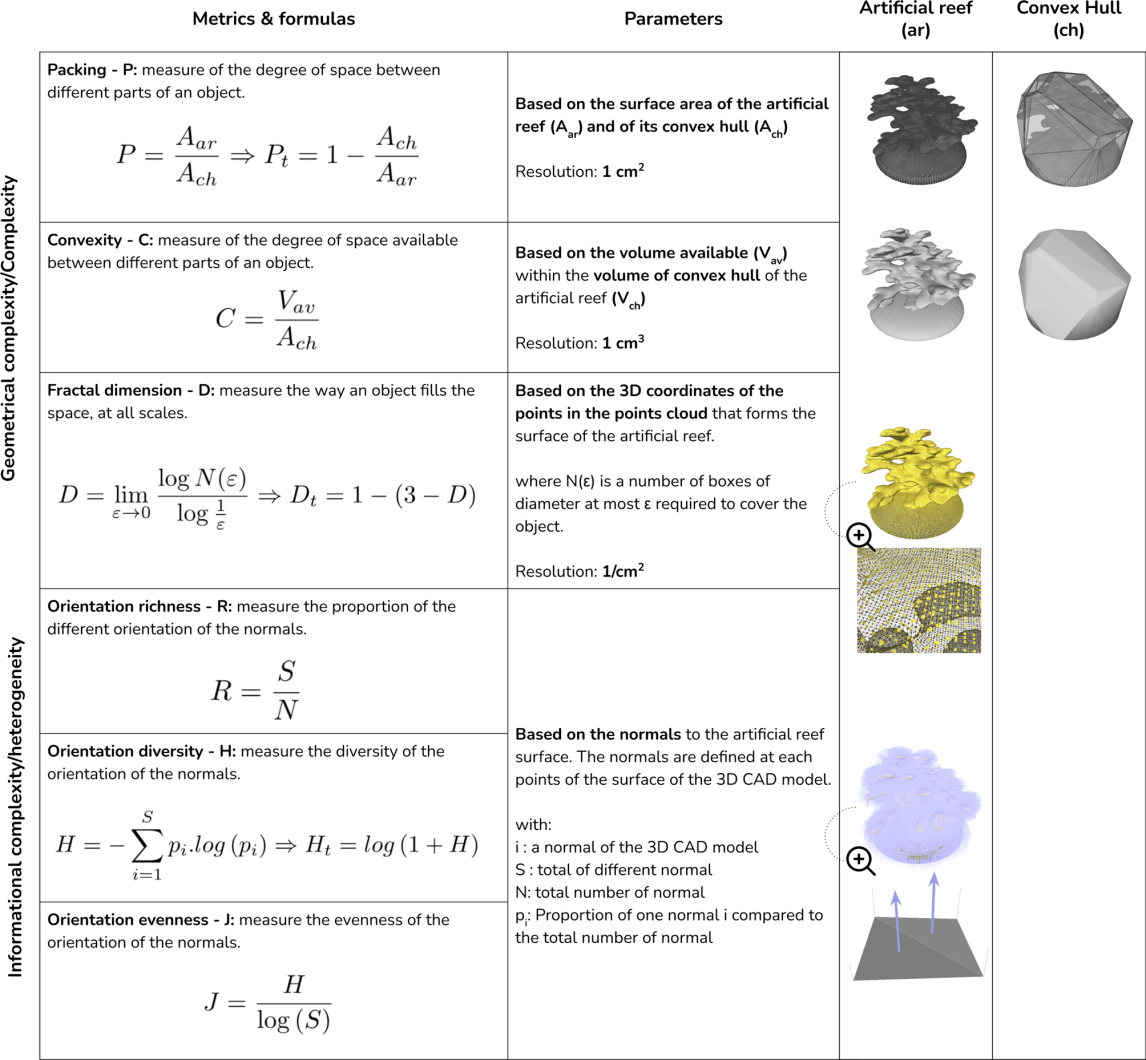
Summary figure providing an overview of the complexity metrics used in the study, which are classified as geometric (3 first rows) and informational (3 last rows). The first column describes the definition and formula for each metric, while the second column lists the parameters used to compute these metrics, including surface, volume, point clouds, and normals. The last columns of the figure include an example of a 3D CAD artificial reef and its convex hull, which illustrate the application of these parameters.

We used Python programming language (version 3.12.1) to extract various parameters from the STL files of the 3D CAD models, such as surface area, volume, point clouds and associated normals (refer to Fig. 1 for details).

All parameters were extracted with a 1 cm resolution to balance computation time and structural definition. When applying this to future biodiversity data, selecting the resolution carefully in advance is crucial to ensure comprehensive and accurate analysis. A 1 cm resolution was chosen because it was suitable for studying both fixed macro benthic communities and mobile species such as fish. Although a finer scale resolution would have been beneficial for monitoring larval and propagule settlement during the early stages of colonization, it would have doubled the fractal dimension computation time for complex structures like 3D-printed models, extending processing time to over 24 h and requiring more memory—resources that might not be accessible to all stakeholders wishing to apply this method. Additionally, the fractal dimension score showed only slight variations between 1 cm and 1 mm resolutions, with the most significant differences observed when transitioning from meter to decimetre scales and from decimetre to centimetre scales.

Using the extracted parameters and elements, we computed most of the metrics with the Python framework (version 3.12.1), utilizing the "entropy" function from the "scipy.stat" package to calculate H.

D was calculated using the Minkowski-Bouligand method (or "box-counting") in the R statistical framework (version 4.0.3) with the “est.boxcount” function from the “Rdimtools” package, which required the computation of point clouds^52^.

To ensure equivalent weight for the variables, we transformed P, D, and H, and named the transformed variables P_t_, D_t_, and H_t_. To summarize the overall complexity of the artificial reefs, we computed an additional metric called the Complexity Index (CI), which is simply the sum of the six metrics. This additional metric provides an overview of the global complexity.

All steps of the computation and details on transformations are summarized in Fig. 1. All the scripts and data are open access (see section Data availability statement).

### Data analyses

#### Multiple factor analysis (MFA)

Statistical analyses were conducted using the open-source software R (version 4.0.3). We performed a Multiple Factor Analysis (MFA) on the indices using the “FactoMineR” package. We grouped the two types of indices (geometric and informational) into separate categorical groups of variables. The Complexity Index (CI) was set as a supplementary quantitative variable and, therefore, was not used to determine the dimensions of the analysis; it was projected onto the existing factor space as an illustration of the global complexity.

#### Clustering

To verify if the purpose categorization was consistent with complexity attributes, we conducted hierarchical clustering on principal components using the “HCPC” function of the “FactoMineR” package. We fixed the number of groups at three, corresponding to protection, production, and bio-mimicry purposes. Finally, the “Catdes” function of “FactoMineR” was used on the Euclidean distance matrix of the scaled complexity variables to describe the clusters.

#### Complexity target points (CTPs)

Based on the Multiple Factor Analysis (MFA) results, we identified 3D models that best represent high complexity to establish Complexity Target Points (CTPs), which summarize the most complex structures evaluated. The selection process focused on the primary axes of variation identified by the MFA and their relationships with the complexity metrics. To guide this selection, we calculated the mean Euclidean distance of the models within the MFA space. This ensured the chosen models were well-represented by the relevant dimensions and exhibited a balanced distribution across the ordination space. The final subset of models was identified by combining their scores on key dimensions with their mean distance metrics. These selected models, representing the highest overall complexity, were then used to calculate the mean values for each complexity variable, which served as the basis for determining the CTPs.

## Results

### Evaluation of the structure of the AR modules

The computed complexity indices for the 3D CAD models of the artificial reefs did not show consistent rankings across all structures. Regarding geometric complexity, the Convexity (C) values ranged from 0.145 (PROD1) to 0.924 (PROT1), transformed Packing (P_t_) values ranged from − 0.031 (PROT5) to 0.765 (BIOM6), and transformed Fractal dimension (D_t_) values ranged from 0.026 (PROT2) to 0.529 (BIOM6). In terms of indices related to informational complexity, the Orientation Richness (R) values ranged from 1.16.10^–6^ (PROT1) to 0.905 (BIOM6), Orientation diversity (H_t_) values ranged from 0.527 (PROT1) to 2.603 (BIOM6), and Orientation Evenness (J) values ranged from 0.414 (PROD7) to 1 (for PROT1, PROT3, PROD5) (Table 1).

**Table 1:** Metrics and Complexity index computed on the artificial reef’s models ordered by their score on the first dimension (Dim. 1) of the of the Multiple Factor Analysis (MFA).

AR's model	C—Convexity	P_t_—Packing	D_t_—Fractal dimension	R—Orientation richness	H_t_—Orientation Diversity	J—Orientation eveness	CI—Complexity index	Dim.1 Score	Dim.1 × Dim.2 distance
PROT5	0.633	− 0.031	0.124	0.000	1.200	0.523	2.449	− 1.729	1.729
PROT1	0.924	0.009	0.081	0.000	0.527	1.000	2.541	− 1.699	2.276
PROT3	0.649	0.108	0.129	0.000	0.527	1.000	2.413	− 1.438	1.697
PROT2	0.922	− 0.026	0.026	0.002	1.780	0.815	3.519	− 1.412	1.853
PROT6	0.262	0.103	0.116	0.001	1.480	0.675	2.637	− 1.223	1.311
PROD1	0.145	0.369	0.249	0.011	1.560	0.450	2.784	− 0.716	1.696
PROD2	0.193	0.394	0.260	0.012	1.620	0.478	2.957	− 0.612	1.560
PROT4	0.761	0.398	0.213	0.001	1.550	0.665	3.588	− 0.550	0.552
PROD7	0.815	0.648	0.352	0.000	1.220	0.412	3.447	− 0.193	0.963
***PROD4***	***0.221***	***0.520***	***0.357***	***0.066***	***1.970***	***0.593***	***3.727***	**0.089**	**1.323**
PROD5	0.564	0.611	0.497	0.000	0.527	1.000	3.199	0.225	0.295
BIOM3	0.382	0.270	0.203	0.658	2.430	0.881	4.824	0.368	0.542
PROD6	0.676	0.608	0.359	0.000	1.760	0.994	4.397	0.499	0.520
BIOM1	0.633	0.362	0.210	0.672	2.440	0.889	5.206	0.598	0.934
BIOM2	0.310	0.369	0.246	0.694	2.470	0.904	4.993	0.677	0.708
BIOM4	0.311	0.388	0.246	0.700	2.480	0.907	5.032	0.744	0.766
***PROD3***	***0.430***	***0.721***	***0.529***	***0.064***	***2.200***	***0.736***	***4.680***	**1.034**	**1.476**
***BIOM7***	***0.624***	***0.451***	***0.304***	***0.539***	***2.500***	***0.988***	***5.406***	**1.035**	**1.193**
***BIOM5***	***0.646***	***0.578***	***0.390***	***0.839***	***2.590***	***0.995***	***6.038***	**1.682**	**1.802**
***BIOM6***	***0.828***	***0.765***	***0.482***	***0.905***	***2.600***	***0.997***	***6.577***	**2.623**	**2.674**
Complexity target points (CTPs)									
MEAN	**0.550**	**0.607**	**0.412**	**0.483**	**2.372**	**0.862**	**5.286**		
SD	**0.232**	**0.133**	**0.092**	**0.405**	**0.277**	**0.187**	**1.123**		

The complexity target points for the metrics and complexity index are calculated as the average values of the selected models. These models were chosen based on their Euclidean distance from the origin in the Dim.1 × Dim.2 space, specifically those with distances greater than or equal to the average distance in this space. Selected models are highlighted in *italic* and bold.

The two first dimensions of the MFA represented 67.48% of total inertia and mainly structured the factor map (Dim.1: 43.21% and Dim.2: 24.28%). These dimensions displayed a good projection of the data, as evidenced by the proximity of all variables to the correlation circle. According to the Karlis-Saporta-Spinakis (KSP) rule^44^ for selecting the number of principal components to retain for the analysis, the third dimension displayed a cumulative (of the two groups of variables) eigenvalue of 0.18, which was below the KSP threshold (2.03). Thus, only the first two dimensions were retained for the analysis.

**Fig. 2:**
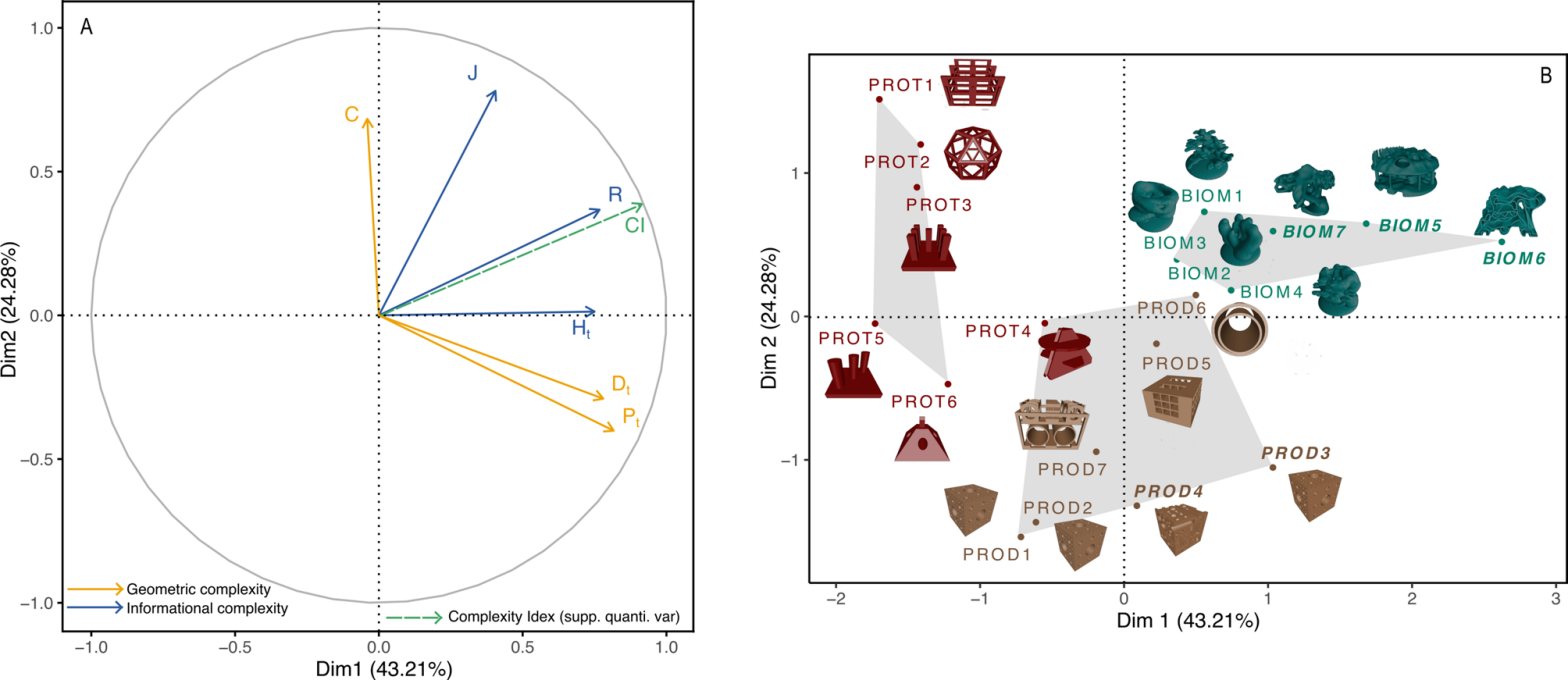
Multiple factor analysis (MFA). (**A**) correlation circle of the variable of complexity coloured according to the type of complexity measurement (i.e. geometric vs informational) and complexity index (CI) as supplementary quantitative variable. (**B**) the score map of the artificial reef models, coloured according to their construction objectives and ordinated according to the clustering. The models selected for the complexity target points are enhanced in *italic* and bold.

H_t_, R, P_t_ and D_t_ contributed equally to building the first dimension (respectively, 22.41%, 21.80%, 26.38%, and 23.64%), while J and C mainly contributed to building the second one (respectively, 38.04% and 25.32%). The Complexity Index, implemented as a supplementary quantitative variable, showed a strong correlation with the first dimension (Pearson’s cor.test Dim.1 vs CI: R = 0.92, t = 10.384, df = 18, p-value = 4.986e-09) and poor correlation with the second (Pearson’s cor.test Dim.2 vs CI: R = 0.35, t = 1.6324, df = 18, p-value = 0.12). (Fig. 2A).

The clustering of the artificial reefs' (AR) 3D CAD models was consistent with the intended purpose of each design. Protective purpose ARs were mostly grouped in Cluster 1, except for PROT 4. This cluster was characterized by negative scores on Dimension 1, influenced mainly by negative scores for P_t_, D_t_, H_t_, and CI (Fig. 2A and B). Cluster 2 included all the production models and PROT 4, described by negative scores on Dimension 2 and metrics J and R but positive scores for P_t_ (Fig. 2A and B). Cluster 3 grouped all the 3D printing models designed for bio-mimicry purposes. These models were positively described by Dimensions and metrics R, H_t_, J, and CI (Fig. 2A and B).

### Selection of 3D models for determining complexity target points (CTPs)

To identify the 3D models that best represent high complexity and establish Complexity Target Points (CTPs), we focused on the first two dimensions of the Multiple Factor Analysis (MFA). We began by calculating the Euclidean distances of the models based on their coordinates in Dim.1 and Dim.2 (Table 1).

Dim.1, the primary axis of variation, captures 43.21% of the total variance and is primarily associated with metrics such as Ht (Orientation Diversity), R (Orientation Richness), Pt (Transformed Packing), and Dt (Transformed Fractal Dimension) (Fig. 2A). These metrics reflect both informational complexity (R and Ht) and geometric complexity (Pt and Dt). The strong correlation between Dim.1 and the Complexity Index (CI) (R = 0.92) suggests that models with high positive scores on Dim.1 likely have higher overall complexity. Although CI was not directly used to construct Dim.1, this correlation supports the use of Dim.1 as a proxy for overall complexity. Consequently, our initial selection focused on models with positive scores on Dim.1.

Dim.2 captures additional variance primarily related to C (Convexity) and J (Orientation Evenness), which are important aspects of complexity not captured by Dim.1 (Fig. 2A). While these metrics are crucial for understanding shape uniformity and volume considerations, they do not dominate overall complexity to the same extent as those in Dim.1. By considering Dim.2, we ensured the inclusion of models that exhibit significant features related to convexity and orientation evenness, thus preventing these ecological aspects from being overlooked (Table 1).

To ensure that models with positive scores on Dim.1 were well represented across both dimensions, we calculated their mean Euclidean distance within the MFA space (mean Euclidean distance for models with positive scores on Dim.1: 1.112 ± 0.689). We used this value as a threshold to select the "complex" models. The selected models are BIOM5 (Distance: 1.802), BIOM6 (Distance: 2.674), BIOM7 (Distance: 1.193), PROD3 (Distance: 1.476), and PROD4 (Distance: 1.323) (Table 1 and Fig. 2B).

We then calculated the mean ± standard deviation for each complexity variable of these "complex" models to establish the Complexity Target Points (CTPs). These CTPs represent the average metrics a 3D model must achieve or surpass to be considered "complex." The CTPs are as follows: CTP-C: 0.555 ± 0.232; CTP-Pt: 0.607 ± 0.133; CTP-Dt: 0.412 ± 0.092; CTP-R: 0.483 ± 0.405; CTP-Ht: 2.372 ± 0.277; J: 0.862 ± 0.187; CTP-CI: 5.286 ± 1.123 (Table 1).

## Discussion

A single metric alone cannot fully capture the complexity of habitat structures^31,34,36,39^. Therefore, we have selected a combination of metrics that, when considered together, provide a comprehensive estimate of the structural complexity across various parameters such as surface area, volume, and point clouds with associated normals extracted from the STL file of the 3D CAD models. To assess all aspects of the structure of the artificial reef models, we based our method on three metrics related to geometric complexity (D_t_, P_t_ and C) and three metrics related to informational complexity (R, H_t_ and J). While we acknowledge that there are potentially infinite mathematical methods to calculate structural complexity and that more advanced mathematical knowledge might yield increasingly complex and potentially more accurate metrics, our focus is within the realms of ecology and engineering. We have deliberately chosen metrics that can be straightforwardly linked to ecological parameters. We believe that introducing overly abstract mathematical formulas would not be practical for our ecological and engineering objectives.

Our methodology aimed to quantitatively assess the geometric and informational complexity of artificial reefs using 3D computer-aided design (CAD) models and propose target points to achieve high complexity of AR’s design prior to immersion to enhance the attraction of diverse and abundant communities theoretically. We proposed a framework that evaluated the global complexity of the structure based on a wide range of artificial reef models, comprising both conventional models for moulding (for protection and production purposes) and bio-mimicry models designed for 3D printing (to enhance biodiversity).

### Surface and volume metrics as basic indicators for assessing ecological suitability of artificial reefs

A suitable substrate is essential for marine benthic organisms, providing the foundation for attachment, growth, movement, and the spread of life from biofilm to epibenthic species^47,53–55^. In habitat complexity literature, surface-derived metrics are frequently employed, the most famous being rugosity^56–61^. The concept of rugosity refers to the refolding aspect of the surface in relation to an orthogonal plan. This parameter was first evaluated through the chain and tape method^61^, which provides a linear measurement of rugosity. However, with the advancements in 3D modelling and reconstruction techniques, it has progressed to encompass 3D surface rugosity^57^ and, more recently, the concept of Packing^41^ has been introduced and successfully used to compare the refolding surface of the coral structure in relation to its convex hull^62^.

While the surface offers vital substrate, the available volume within the habitat structure provides the necessary physical space for organisms to move and carry out their life processes: it provides shelter to survive, reproduce, or maintain their ecological roles^63^. Volume metrics are less commonly used in habitat complexity studies, likely due to the challenges in evaluating them in a natural environment^64^. More recently, thanks to tomography or scanner technology, volume-driven metrics can be computed from 3D CAD models of habitat fragments, such as coral^62,65,66^. Assessing volume parameters becomes easy using the metrics Convexity introduced with Packing by Zunic and Rosin^41^.

### Incorporating fractal and surface orientation metrics in habitat evaluation: addressing multiscale complexity

Habitats are inherently multiscale and provide a diverse range of microhabitats that meet the needs of different life stages and ecological roles of organisms^42–44,67^. From primary producers to predators, it supports a broader range of species and ecological interactions, providing a rich food web for biodiversity^42–44^ and resilience to environmental stressors^68,69^. An artificial reef is expected to provide various microhabitats at different scales to support a diverse and abundant community. Thus, we used the fractal dimension to measure how an object fills space at different scales. It has been widely used in marine ecology to describe the relationship between species diversity and the structure of different marine habitats, such as coral reefs, seagrass beds, and rocky intertidal zones^34^. Nowadays, it is even easier to compute it on habitat reconstruction with 3D CAD modelling by photogrammetry or 3D scanning^59,66^. We have been cautious in choosing a resolution to compute the fractal dimension relevant to our study case (1 point/cm^2^). Our goal was to achieve a balance between computation time and structural clarity, thereby excluding finer details. We are confident this resolution will satisfy our objectives, focusing on benthic macrofouling and mobile species.

We based our evaluation of the informational complexity of the artificial reef models on the distribution of normal vectors. This gives information on the surface orientation of the structure, which is critical for both fixed and mobile marine species. For fixed species, such as corals, sponges, and algae, surface orientation affects their ability to capture light, nutrients, and planktonic prey (for coral and sponge), essential for their survival and growth^54,55,70,71^. The orientation can also influence their ability to resist physical disturbances such as strong water currents or waves^72^. For mobile species, surface orientation provides shelter and plays a crucial role in the ability of species to navigate, detect prey, and avoid predators^73^. Overall, surface orientation is an important factor affecting marine species’ distribution, abundance, diversity, and interactions with each other and their environment.

Moreover, the normal vector is the only parameter whose heterogeneity can be quantified without relying on subjective observations, such as manually counting shelters or cavities. While these techniques can estimate informational complexity, they are often impossible or time-consuming. Counting micro-habitats on a model is particularly difficult for several reasons. First, the scale of the community targeted—whether fixed or mobile—affects how micro-habitats are determined. Complex designs, such as bio-mimicry models with interconnected shapes, make it even harder to identify and count micro-habitats objectively. Except for some obvious cavities, the rest remain highly subjective.

Therefore, we support using normal vectors as a parameter in our study. Existing metrics use the normal parameters^30,36,59,74–76^, offering diverse values to identify surface topography (related to geometric complexity): strength vector, vector dispersion, and several standard deviations to the plane. We used metrics commonly applied in ecology to determine habitat informational complexity derived from Webb and colleagues^41^, Shannon^42^ and Pielou^43^ indexes, named respectively in our study: orientation Richness (R), Orientation diversity (H_t_), and Orientation evenness (J). We used these indexes to assess habitat informational complexity as a proxy of the potential diversity that the reef can welcome. These metrics provide information on the proportion of the different types of surface orientations, their diversity in relation to their relative abundance and distribution.

### Scaling up: proposing complexity target points to assess the ecological potential of artificial reefs

With the six metrics selected, we embrace global complexity (both geometrical and informational) by evaluating variations in surface, volume, scale, types of elements, and their relative abundance.

Using multifactorial analysis, we identified Complexity Target Points (CTPs) by selecting models with the highest complexity features scores. It is important to consider that the proposed targets represent optimal complexity levels based on a sample of artificial structures. However, environmental factors, such as depth, current, light, and connectivity to surrounding adjacent habitats, influence community responses^77–80^. Therefore, while the CTPs are grounded in habitat complexity theory^36,81–85^ and suggest that structures meeting these targets should attract a diverse and abundant community, the actual community composition may vary due to other additional environmental factors. Therefore, relying solely on these proposed targets might underestimate the ecological responses when an artificial structure is deployed in a natural environment.

To delve deeper into the geometrical complexity metrics and the associated CTPs, we start with C. This metric stands out as the sole volume-based measure, contrasting with other geometric complexity metrics derived from surface properties. Interpretation of C values is nuanced: a value close to 1 indicates an empty structure devoid of hiding places from predators, whereas a value near 0 signifies a filled structure with no cavities available for shelter. The computed CTP for C reflects a moderate value (CTP-C: 0.550 ± 0.232), striking a balance between space availability and structural integrity, providing enough living space necessary to attract an abundant and rich community^63^. For the other metrics, transformed Packing and transformed fractal dimension also demonstrate moderate values (CTP-P_t_: 0.607 ± 0.133, CTP-D_t_: 0.412 ± 0.092), reflecting a straightforward logic. A highly folded surface correlates with a high transformed fractal dimension (cor.test P_t_ vs D_t_: R = 0.93, t = 10.463, df = 18, p-value = 4.432e−09), as seen in the MFA results (Fig. 2). Therefore, a structure with a score approaching 1 for P_t_ or D_t_ would result in a surface object with structural elements of habitat becoming too small to be beneficial for organisms of any size^34^. In nature, mechanisms or organisms that exhibit a certain fractality are governed by rules acting at both small and large scales, in both ascending and descending manners, thus limiting the extent of their fractality^67^. The CTP of the metrics P_t_ and D_t_, which are strongly correlated, reflect this limitation.

Turning to informational complexity and the associated CTPs, we begin with J, which measures the equitability of normal vectors’ distribution. As the C metric, J is not straightforward to understand; both simple and complex structures can display a high score (i.e.: 1). Euclidean shapes such as a cube, a sphere or a triangle, for instance, display the highest score because each face of these shapes is oriented differently. Therefore, J should be considered alongside the other informational complexity metrics to provide a comprehensive assessment of the reef structure. If R or H_t_ are below their respective CTP (CTP-R: 0.483 ± 0.405; CTP-H_t_: 2.372 ± 0.277) while J is high, it means that we have a simple structure, in the reverse case, low J but high R and H_t_, it means then that the orientation of the structure is dominated by few different normal vectors. Consistently, the CTP-J should preferably be high (0.862 ± 0.187) to propose a structure with an equitable distribution of different surface orientations.

Building on this analysis of complexity metrics, our method proved valuable in evaluating the three purposes of the artificial reef structures based on their complexity metrics. This validates the empirical approaches used historically, confirming their relevance and accuracy, considering our current results. From protection to bio-mimicry, the three categories were perfectly distributed along the first dimension of the MFA. Protection designs, with the simplest shapes and most voided space, had the lowest Complexity Index scores (CI.mean = 2.858 ± 0.545, C.mean = 0.692 ± 0.246, P_t_.mean = 0.094 ± 0.161, D_t_.mean = 0.115 ± 0.062, R.mean = 0.001 ± 0.001, H_t_.mean = 1.177 ± 0.537, J.mean = 0.780 ± 0.194). These structures are not intended to attract rich communities, so their low scores do not detract from their purpose of habitat protection. In contrast, biomass production structures, more massive and complex, showed lower C but higher P_t_ and D_t_ scores and globally low informational complexity scores (CI.mean = 3.599 ± 0.717, C.mean = 0.435 ± 0.261, P_t_.mean = 0.553 ± 0.132, D_t_.mean = 0.372 ± 0.107, R.mean = 0.022 ± 0.030, H_t_.mean = 1.551 ± 0.548, J.mean = 0.666 ± 0.250). Bio-mimicry designs exhibit the highest scores for the Complexity Index and all informational complexity metrics, but moderate values of geometrical metrics (CI.mean = 5.335 ± 0.426, C.mean = 0.505 ± 0.178, P_t_.mean = 0.430 ± 0.090, D_t_.mean = 0.279 ± 0.071, R.mean = 0.689 ± 0.107, H_t_.mean = 2.496 ± 0.057, J.mean = 0.937 ± 0.051).

Nowadays, in the context of the reconciliation ecology, artificial structures are intended to exhibit several functions at the same time, such as sea level rise mitigation in combination with bio-mimicry to become a “grey nature-based solution” when green ones cannot be applicable^86^. Therefore, designers can evaluate new artificial structures before deployment using this method and framework provided in this study. This process helps identify specific areas for improvement and optimizes design characteristics such as increasing space availability, enhancing surface refolding, varying shelter scales, or increasing the heterogeneity of surface orientations. These enhancements aim to improve the ecological performance of the structure, ensuring it meets the Complexity Target Points (CTPs).

By providing a clear framework for establishing Complexity Target Points (CTPs), we aim to offer practical guidelines for future artificial structure design that seek to enhance colonization performance by optimizing complexity metrics. Recognizing that our current analysis is foundational, we propose the following approach to substantiate the ecological benefits of our method. Although the lack of post-immersion data is a limitation to validating our CTPs, this can be mitigated through in situ pilot studies to monitor the actual biodiversity and community distribution in correlation with the CTPs of the studied reef and make the CTPs more specific for certain species of interest. Another perspective involves comparing the different metrics’ scores of bio-mimicry artificial structures with those obtained from 3D reconstructions of natural reefs made by photogrammetry^87–92^. This comparison would be valuable for advancing the CTPs for bio-mimicry efforts and aligning artificial structures more closely with the metrics of natural habitats.

## Conclusion

We argue that our approach, which focuses on the structural aspects of artificial reefs, can contribute to the development of global artificial reef design and support ecological reconciliation and restoration efforts by enhancing landscape complexity in the face of growing marine artificialization and habitat degradation^93–95^. In conjunction with the methodology developed by Carral and colleagues^96^, which considers other extrinsic parameters such as stakeholder engagement and immersion site selection, the effectiveness of artificial reef deployment projects may be, nowadays, enhanced by a more rigorous scientific framework.

## Supplementary Information

Supplementary Information.

## Data Availability

The data and code for this research are openly accessible on Zenodo and GitHub: 10.5281/zenodo.8091788, https://github.com/ELI-RIERA/ArtificialReef_Complexity. However, please note that the 3-dimensional computer-aided design (3D CAD) models, proprietary to BOSKALIS, D-SHAPE and SEABOOST, are not included. For access to these specific 3D CAD models, contact the corresponding author.
